# The Chest Wall Mass of Unknown Origin: A Rare Presentation of Esophageal Adenocarcinoma

**DOI:** 10.7759/cureus.59209

**Published:** 2024-04-28

**Authors:** Arcole Brandon, Karan B Singh, William Sonnier

**Affiliations:** 1 College of Medicine, University of South Alabama, Mobile, USA; 2 Internal Medicine, University of South Alabama, Mobile, USA; 3 Gastroenterology and Hepatology, University of South Alabama, Mobile, USA

**Keywords:** stage iv cancer, chest wall, esophagus, esophageal mass, esophageal cancer, unexpected metastasis, chest wall mass, esophageal adenocarcinoma

## Abstract

A chest wall mass can result from a diversity of underlying disease processes ranging from benign to a site of distant metastasis. The chest wall is a rare site for esophageal adenocarcinoma (EAC) metastasis. Delayed diagnosis can occur when presenting symptoms are not typical of esophageal pathology, and advanced-stage EAC has a high morbidity and low survival rates. Our case demonstrates a rare and unusual presentation of EAC with a poor outcome due to delayed diagnosis.

## Introduction

Esophageal cancer is the eighth most common cancer worldwide, and it accounts for over 500,000 deaths annually [1]. Esophageal cancer is usually either an esophageal squamous cell carcinoma (ESCC) or an esophageal adenocarcinoma (EAC). ESCC holds a higher prevalence globally, but EAC is more common in the USA. Barrett’s esophagus, a precursor lesion, is the leading risk factor for EAC [1,2].

Clinically, esophageal cancer is an occult disease and initially asymptomatic, which can lead patients to delay seeking medical attention. Typical presentations include dysphagia or odynophagia, worsening fatigue, and weight loss. Less commonly, patients may present with hoarseness from recurrent laryngeal nerve injury and melena from tumoral bleeding [2]. When diagnosed early, EAC has a cure rate of approximately 50% with surgical resection, but it has an overall high morbidity and a 15% survival rate over five years [3]. EAC commonly metastasizes to the liver and lungs. Other sites are much less common, including the chest wall, which is precisely what is demonstrated in our patient’s case [4-6].

## Case presentation

A 56-year-old male with no known medical history other than obesity presented to the hospital with several weeks of progressively worsening right shoulder pain radiating throughout his back. The patient denied any history of cigarette smoking and illicit drug use, but he endorsed his father having an unknown lymphoma and his mother having an unknown gynecologic cancer that resulted in her death. Upon further questioning, he reported a five-year history of dysphagia that recently worsened, vomiting, melena, night sweats, and an unintentional 40-pound weight loss over the prior four months. The patient had previously undergone medical management for his symptoms with pantoprazole and sucralfate but never had any prior imaging studies or further workup. A physical exam revealed pain with palpation over the right chest wall and paraspinal muscles of the cervical and thoracic spine with a reduced range of motion in the right upper extremity. Vital signs were normal. His admission labs were significant for the following values as presented in Table 1.

**Table 1:** Notable Admission Lab Values Na, sodium; Alb, albumin; ALP, alkaline phosphatase; ALT, alanine aminotransferase; AST, aspartate aminotransferase; GGT, gamma-glutamyl transferase; LDH, lactate dehydrogenase; Hb, hemoglobin; Hct, hematocrit

Tests	Results	Reference Ranges
Na	131 mmol/L	136-145 mmol/L
Alb	2.9 g/dL	3.4-5.0 g/dL
ALP	430 U/L	45-117 U/L
ALT	60 U/L	14-59 U/L
AST	88 U/L	15-37 U/L
GGT	519 U/L	15-85 U/L
LDH	627 U/L	87-241 U/L
Hb	11.2 g/dL	13.5-17.5 g/dL
Hct	34.9%	41-53%

**Figure 1:**
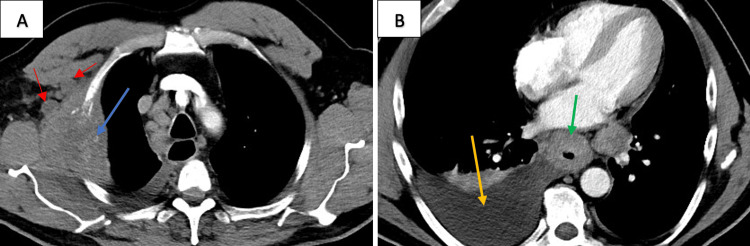
A: Chest Wall Mass; B: Esophageal Wall Thickening A. Large (7.7x5.0 cm) destructive soft tissue mass with bone erosion involving the right second rib in the costal-pleural region (blue arrow) with surrounding lymphadenopathy (red arrows) B. Diffuse, circumferential wall thickening of the thoracic esophagus (green arrow) with a moderate right pleural effusion (orange arrow).

**Figure 2:**
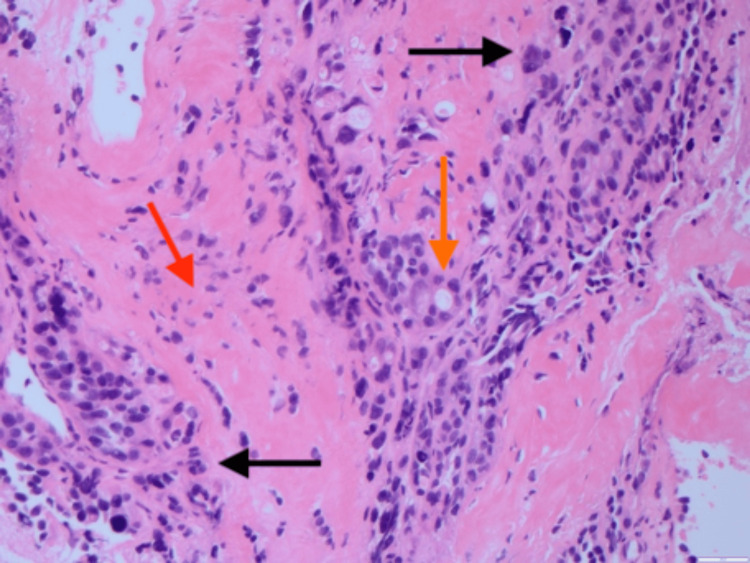
Poorly Differentiated Adenocarcinoma of Unknown Origin H&E stain (magnification: 20x) of the chest wall biopsy demonstrating malignant epithelial cells with pleomorphism, hyperchromasia, nuclear irregularities (black arrows), glandular differentiation (orange arrow), and infiltrating tumor cells in a desmoplastic fibrotic stroma (red arrow) concerning for an adenocarcinoma of unknown origin.

**Figure 3:**
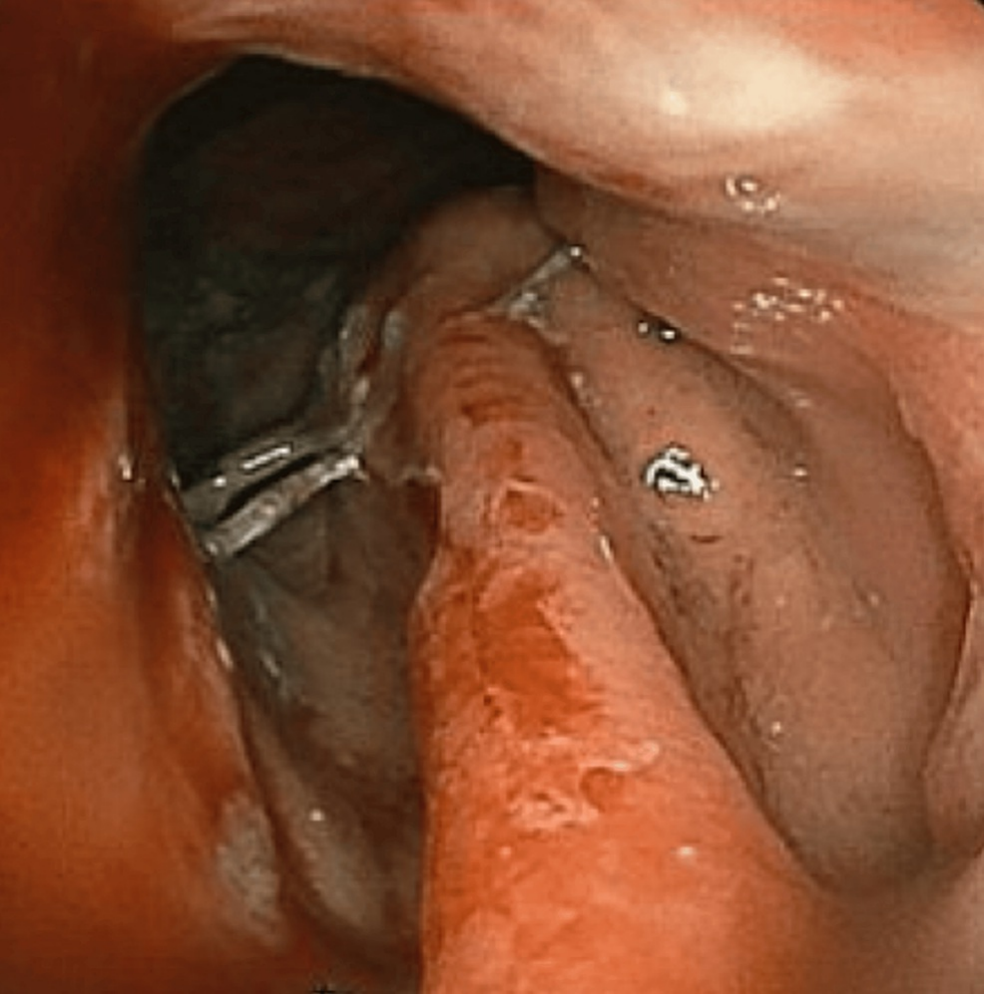
Ulcerated Esophageal Mass EGD demonstrating a large friable, ulcerated mass in the distal esophagus that was noted to be present from the gastroesophageal junction at 42 cm to 29 cm from the incisor teeth. EGD, esophagogastroduodenoscopy

Computed tomography (CT) of the thorax with contrast showed a large destructive lesion involving the right second rib with further suspicious lesions throughout the bilateral lungs and circumferential wall thickening of the esophagus (Figures 1A, 1B). Further imaging demonstrated suspected metastatic disease in the liver, lumbar and thoracic spine, and occipital lobe. CT-guided biopsy of the right chest wall mass demonstrated a poorly differentiated adenocarcinoma of unknown origin (Figure 2). Esophagogastroduodenoscopy (EGD) demonstrated a large friable, ulcerated mass (Figure 3). The EGD’s biopsy results were positive for EAC. After the patient was stabilized and the oncologic plan was established, he was discharged and underwent chemotherapy and immunotherapy, which was complicated by drug reaction with eosinophilia and systemic symptoms (DRESS) syndrome, before passing away five months later. 

## Discussion

Our case demonstrates a rare presentation of EAC with metastasis to the chest wall that adds to the few reported cases in the literature [3-4,7-10]. While EAC commonly metastasizes to the liver and lungs, there are other locations to which it only rarely metastasizes, such as the subcutaneous region. A review from a single oncology center demonstrated only three out of 102 EAC metastases to the subcutaneous region [11]. While being located directly below the skin is an infrequent site of EAC metastasis in general, it is furthermore evident that the chest wall specifically is a very rare anatomic location of EAC subcutaneous metastatic disease. A systematic review showing the locations of 164 unexpected esophageal cancer metastases, of which 39.5% were EAC and 60.5 % were ESCC, demonstrated only two cases of esophageal cancer that were metastatic to the chest wall. Regardless of the histological subtype, metastasis to the chest wall is evidently very rare for primary esophageal cancer [6].

Chest wall metastasis from EAC occurs either from lymphohematogenous spread or from the embedding of tumor cells into the chest wall during surgical resection of the primary tumor [4]. Our patient’s cancer likely spread to his chest wall via a lymphohematogenous route as he had no knowledge of his underlying EAC. In a reported case with a prior esophagectomy, the chest wall mass was the marker of recurrence [7]. In our patient’s case, the chest wall mass was the initial presentation of EAC metastasis as opposed to a marker of recurrence. 

Generally, chest wall masses are either benign hyperplasia of local soft tissue, such as a lipoma, malignant transformation of underlying tissue, such as chondrosarcoma, or implants from metastasis [12]. In a previously reported case, a patient was found to have a metastatic chest wall implant after he electively requested to have a non-tender lump on his back resected at the time of his esophagectomy, which was found to be an EAC metastatic lesion [8]. That case demonstrates how EAC metastasis to the chest wall can be asymptomatic, but as our case and other prior cases demonstrate, EAC metastatic chest wall lesions can be acutely painful [4,7]. The cause of this pain is likely multifactorial in our patient's case, specifically due to bone invasion and associated nerve impingement from the significant size of the mass. This further underscores the importance of early diagnosis as our patient had a five-year history of dysphagia, yet he only previously received medical management for this symptom. The pain from his metastatic EAC chest wall mass was what caused him to present to the hospital and receive a workup for the source of this pain. The failure to recognize this patient's EAC red flag symptoms earlier in the course of the disease led to a significant progression in his disease burden and distant metastasis, which thereby led to his initial presentation to the hospital to seek treatment for the pain associated with his metastatic chest wall lesion.

Metastatic implants to the chest wall portend a poor prognostic sign, and one study found that these have a median survival of five months [13]. Early stage EAC, which is defined here as stage IIA (T2N0M0) or less, has a survival rate of approximately 50% after surgical resection, but EAC patients in stage IVB, which is defined as having metastasis beyond the cervical lymph nodes, have a median survival of under one year [3,8]. Typically, primary breast or lung cancers metastasize to the chest wall [12]. However, there can be unexpected metastasis from other organs, such as the esophagus and thyroid [14]. As these cases demonstrate, a chest wall mass can represent atypical findings, and a broad differential diagnosis should be considered in order to make the correct diagnosis. 

## Conclusions

EAC is a form of cancer with a high mortality rate if it is left untreated. While many patients present with classic symptoms, such as dysphagia, if EAC is not diagnosed for an extended period of time, like in the case of our patient, then distant metastatic spread can lead to unexpected symptoms, such as musculoskeletal pain. Our patient originally presented to the hospital for shoulder pain and was found to have a chest wall mass of an unknown origin, but further patient history revealed an extended period of classic red flag EAC symptoms, such as dysphagia and weight loss. It is evidently very rare for an EAC to metastasize to the chest wall, and this patient’s atypical presentation is likely a result of his significant, long-standing cancer burden.

When patients present with a chest wall mass, the differential diagnosis includes many benign conditions and also many malignant pathologies. A chest wall mass should not be ignored as it is rarely the presenting symptom of metastatic cancer, like the case of our patient. A delayed diagnosis can lead to further metastatic spread when the presenting symptoms are not typical of the underlying primary cancer. It is essential that providers take a thorough history as other symptoms can be discovered in this manner as was seen in our patient's case with a history of long-standing dysphagia, a red flag symptom for esophageal cancer. Considering a broad differential diagnosis for a chest wall mass allows physicians to ensure they are not overlooking a presenting symptom of an underlying pathology with a high mortality rate, such as EAC.
